# Ethics and Administration

**Published:** 1894-08-25

**Authors:** 


### ETHICS AND ADMINISTRATION.

### Resident Medical Officer and Matron.

(1) *Should the matron of a hospital have her meals with the nurses, resident medical officers, or alone in her own apartments?* (2) *Under what conditions should the matron and resident medical officers have the privilege of inviting guests to dinner, &c.?* (3) *Would you consider* 13 *guests entertained in one week by one official to be an abuse of the funds of a charity?*

(1) It is usual for the nurses of a large hospital to reside in a separate home. The matron is usually present and often presides at the nurses' dinner. Sometimes she dines alone and usually takes her meals in her own apartments. At some of the county hospitals the old practice of the matron, resident medical officers having their meals together still prevails, but it is happily dying out. (2) The custom is to permit this privilege, the good sense of the officers rendering any regulations unnecessary. If it is abused the committee can suspend the privilege or make such regulations as circumstances may render necessary. (3) We should certainly consider this an abuse of the funds of a charity unless there were extraordinary circumstances to warrant an exception being made.

### Fares and Expenses of Selected Candidates at Elections.

*A Secretary writes: A selection of three is made out of a number of candidates for the post of resident medical officer. The three selected candidates are invited to appear before the Election Committee, their fare* (*second-class return*) *and half-a-guinea towards hotel expenses being allowed to enable them to do so. A copy of the rules is sent for their guidance, and they all agree to attend the committee on the appointed day. They come down the night before, and one immediately on his arrival goes to the hospital, makes certain inquiries, and on the morning of the election fails to appear before the Election Committee, but writes them that, "Since accepting the invitation to appear as selected candidate for the post, he finds on inquiry that the duties are so different from those to which he has been accustomed that he fears he should be unable to carry them out to the satisfaction of the committee, and he therefore asked to be allowed to withdraw his application." This candidate, although he fails to appear before the committee, requests the payment of his railway fare and hotel expenses. Without prejudice, do you consider he is entitled to claim his expenses under the circumstances?*

The expenses and railway fare were offered conditionally upon the candidate's appearance before the committee. As he did not appear, but voluntarily withdrew his application before the committee met, he is not, in our view, entitled to his expenses, and it would be an act of generosity for any committee to pay them in such circumstances.

